# Supplementary material for: Metabolomics Characterizes the Effects and Mechanisms of Quercetin in Nonalcoholic Fatty Liver Disease Development
Source: Int J Mol Sci. 2019 Mar 11;20(5):1220. doi: 10.3390/ijms20051220 (PMC6429195; doi:10.3390/ijms20051220)
Supplement: Supplementary file 1 [file ijms-20-01220-s001.pdf]

Table S1. Effect of quercetin on serum biochemical parameters of rats with NAFLD (Mean±SD).

Parameters	TG (mmol/L)	TC (mmol/L)	LDL (mmol/L)	HDL (mmol/L)	AST (IU/L)	ALT (IU/L)
C30	0.25 ± 0.02	1.95 ± 0.2	0.72 ± 0.04	0.51 ± 0.03	155.23 ± 11.54	43.66 ± 3.06
M30	0.65 ± 0.04	5.47 ± 0.35	2.15 ± 0.19	0.35 ± 0.02	270.12 ± 15.28	97.24 ± 4.44
Q30	0.49 ± 0.03	2.91 ± 0.19	1.22 ± 0.09	0.43 ± 0.02	218.31 ± 13.45	66.29 ± 4.5
C50	0.29 ± 0.02	2.08 ± 0.21	0.77 ± 0.04	0.47 ± 0.03	174.89 ± 11.98	56.12 ± 5.24
M50	0.49 ± 0.04	4.12 ± 0.48	1.93 ± 0.14	0.33 ± 0.02	309.42 ± 22.02	139.78 ± 7.43
Q50	0.42 ± 0.03	2.82 ± 0.22	1.12 ± 0.08	0.42 ± 0.02	270.89 ± 12.94	116.56 ± 6.15

Table S2. Potential differential metabolites detected by HPLC-QTOF-MS among control, model and quercetin groups in 30 days.

Different Metabolites	RT	Mass	C30 vs. M30		M30 vs. Q30	
			VIP	*p* Value	VIP	*p* Value
Adrenic acid	18.800	331.264	1.564	0.002	1.723	0.004
Docosahexaenoic acid	16.777	327.233	1.683	0.002	1.890	0.002
Linoleic acid	17.295	279.233	1.369	0.004	<1	>0.05
Eicosapentaenoic acid	15.768	301.217	1.506	0.002	1.597	0.015
Oleic acid	18.924	281.249	1.506	0.002	1.753	0.004
Palmitic acid	18.463	255.233	1.732	0.002	1.908	0.002
p-Cresol sulfate	7.159	187.007	1.435	0.002	<1	>0.05
Indoxyl sulfate	6.490	212.002	1.529	0.002	<1	>0.05
2-Hydroxybutyric acid	1.579	103.040	1.647	0.002	1.807	0.002
Arachidonic acid	17.077	303.233	1.576	0.002	1.844	0.002
12(S)-HPETE	10.713	335.223	1.686	0.002	1.0012	0.015
Chenodeoxycholic acid glycine conjugate	10.061	448.307	1.703	0.002	1.471	0.026
Taurocholic acid	8.117	514.284	1.667	0.002	<1	>0.05
Glycocholic acid	9.064	464.301	1.695	0.002	<1	>0.05
Citric acid	1.109	191.020	1.684	0.002	1.860	0.004
Succinic acid	1.495	117.019	1.470	0.004	<1	>0.05
15(S)-Hydroxyeicosatrienoic acid	13.574	321.243	1.715	0.002	1.941	0.002
Alpha-dimorphecolic acid	12.460	295.228	1.735	0.002	1.940	0.004
9,10,13-TriHOME	8.968	329.233	1.746	0.002	2.014	0.002

Statistical significance levels were determined by Mann-Whitney *U* test. Only metabolites with *p*-values < 0.05 and VIP > 1 were deemed to be statistically significant.

Table S3. Potential differential metabolites detected by HPLC-QTOF-MS among control, model and quercetin groups in 50 days.

Different Metabolites	RT	Mass	C50 vs. M50		M50 vs. Q50	
			VIP	*p* Value	VIP	*p* Value
Docosahexaenoic acid	16.777	327.233	1.507	0.004	<1	>0.05
Palmitic acid	18.463	255.233	1.661	0.002	1.998	0.041
p-Cresol sulfate	7.159	187.007	1.726	0.002	<1	>0.05
Indoxyl sulfate	6.490	212.002	1.704	0.002	<1	>0.05
2-Hydroxybutyric acid	1.579	103.040	1.478	0.004	1.925	0.041
Arachidonic acid	17.077	303.233	1.265	0.009	<1	>0.05
12(S)-HPETE	10.713	335.223	1.855	0.002	3.015	0.002
12-HETE	13.108	319.228	1.202	0.026	<1	>0.05
Taurocholic acid	8.117	514.284	1.766	0.015	<1	>0.05
Glycocholic acid	9.064	464.301	1.791	0.026	<1	>0.05
Chenodeoxycholic acid glycine conjugate	9.082	448.307	1.785	0.026	<1	>0.05
Succinic acid	1.495	117.019	1.609	0.002	<1	>0.05
Citric acid	1.109	191.020	1.602	0.002	<1	>0.05
L-Tyrosine	1.478	180.067	1.128	0.026	<1	>0.05
15(S)-Hydroxyeicosatrienoic acid	13.574	321.243	1.843	0.002	<1	>0.05
Alpha-dimorphecolic acid	12.460	295.228	1.800	0.002	<1	>0.05
9,10,13-TriHOME	8.968	329.233	1.811	0.002	<1	>0.05

Statistical significance levels were determined by Mann-Whitney *U* test. Only metabolites with *p*-values < 0.05 and VIP > 1 were deemed to be statistically significant.

Table S4. Effect of quercetin on differential metabolites of rats with NAFLD (Mean±SD).

Metabolites	C30	M30	Q30	C50	M50	Q50
Adrenic acid	1.21 ± 0.22	0.62 ± 0.09	0.91 ± 0.14	1.30 ± 0.26	1.04 ± 0.08	0.89 ± 0.18
Docosahexaenoic acid	1.19 ± 0.2	0.44 ± 0.08	0.98 ± 0.24	1.17 ± 0.07	0.90 ± 0.13	1.02 ± 0.15
Palmitic acid	1.29 ± 0.2	0.53 ± 0.08	1.08 ± 0.23	1.27 ± 0.13	0.76 ± 0.11	1.06 ± 0.30
Linoleic acid	1.14 ± 0.2	0.78 ± 0.13	0.94 ± 0.13	1.12 ± 0.11	0.98 ± 0.09	1.04 ± 0.17
Oleic acid	1.17 ± 0.2	0.75 ± 0.10	1.01 ± 0.11	1.14 ± 0.10	0.99 ± 0.13	1.03 ± 0.17
Eicosapentaenoic acid	1.14 ± 0.2	0.61 ± 0.11	0.90 ± 0.18	1.14 ± 0.20	0.99 ± 0.16	0.98 ± 0.17
p-Cresol sulfate	0.59 ± 0.1	1.05 ± 0.21	0.81 ± 0.13	0.54 ± 0.07	1.36 ± 0.25	1.24 ± 0.24
Indoxyl sulfate	0.64 ± 0.08	1.03 ± 0.16	0.92 ± 0.17	0.62 ± 0.09	1.21 ± 0.14	1.06 ± 0.16
2-Hydroxybutyric acid	1.22 ± 0.13	0.68 ± 0.11	1.20 ± 0.25	1.32 ± 0.23	0.82 ± 0.11	0.97 ± 0.14
12(S)-HPETE	0.35 ± 0.04	1.17 ± 0.19	0.98 ± 0.24	0.32 ± 0.06	1.52 ± 0.12	1.08 ± 0.12
12-HETE	0.90 ± 0.18	0.88 ± 0.17	0.90 ± 0.17	0.83 ± 0.16	1.15 ± 0.21	1.02 ± 0.17
Arachidonic acid	1.10 ± 0.15	0.65 ± 0.08	1.10 ± 0.15	1.10 ± 0.09	0.96 ± 0.06	1.03 ± 0.12
Taurocholic acid	0.43 ± 0.08	1.25 ± 0.19	0.43 ± 0.08	0.40 ± 0.06	1.14 ± 0.16	1.09 ± 0.19
Glycocholic acid	0.15 ± 0.02	1.31 ± 0.24	0.15 ± 0.02	0.17 ± 0.03	1.08 ± 0.20	1.09 ± 0.15
Chenodeoxycholic acid glycine conjugate	0.23 ± 0.04	1.34 ± 0.26	0.23 ± 0.04	0.27 ± 0.03	0.97 ± 0.16	1.05 ± 0.13
Citric acid	1.48 ± 0.22	0.54 ± 0.10	1.48 ± 0.22	1.47 ± 0.24	0.82 ± 0.15	1.01 ± 0.14
Succinic acid	0.71 ± 0.10	1.12 ± 0.17	0.71 ± 0.10	0.61 ± 0.13	1.14 ± 0.18	1.14 ± 0.23
L-Tyrosine	1.10 ± 0.11	0.93 ± 0.13	1.06 ± 0.15	1.10 ± 0.03	0.94 ± 0.14	0.99 ± 0.19
Alpha-dimorphecolic acid	0.23 ± 0.04	1.45 ± 0.21	0.23 ± 0.04	0.17 ± 0.03	1.21 ± 0.21	1.21 ± 0.20
15(S)-Hydroxyeicosatrienoic acid	0.21 ± 0.04	1.49 ± 0.27	0.21 ± 0.04	0.19 ± 0.02	1.27 ± 0.17	1.12 ± 0.14
9,10,13-TriHOME	0.16 ± 0.03	1.64 ± 0.23	0.16 ± 0.03	0.17 ± 0.03	1.22 ± 0.21	1.25 ± 0.18

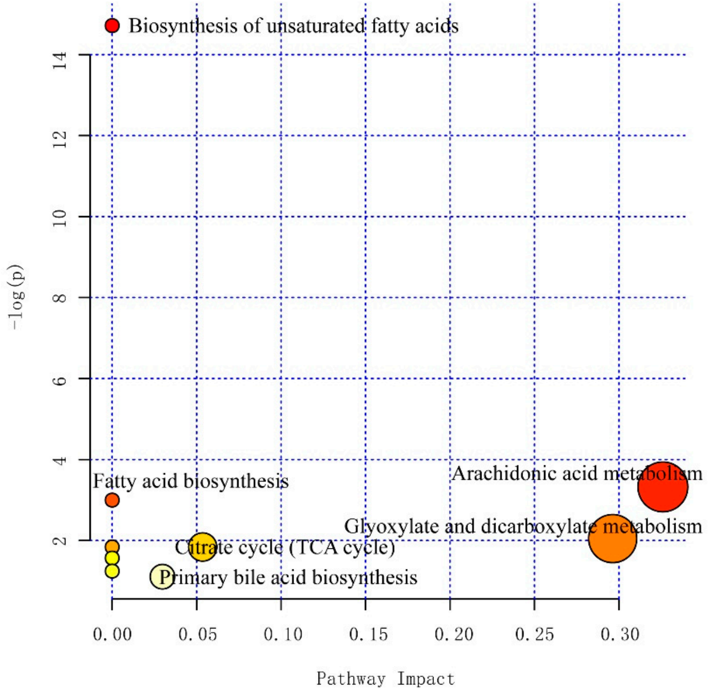

Figure S1. Quercetin-targeted pathways in rats with NAFLD in 30 days.
